# Internal carotid artery dissection: An unusual cause of occipital infarction

**DOI:** 10.4103/0972-2327.64634

**Published:** 2010

**Authors:** Inuka K. Gooneratne, Ranjanie Gamage, Kamal S. Gunarathne

**Affiliations:** National Hospital, Sri Lanka

Carotid artery dissections (CAD) are uncommon but not rare. Incidence studies indicate between 2.5 and 3 cases per 100 000 population for all age groups, mean age of onset in the early 40s.[1] Traumatic carotid artery dissection is a recognized complication following severe head injury and trauma to the neck. CAD is an identified cause of stroke and accounts for 20% of strokes in the young.[2] Incidence of stroke before the age of 40 years accounts to nearly 10% of all strokes in the South Asian region.[3] Internal carotid artery dissections usually produce stroke in the middle cerebral artery territory or the border zone between the middle and anterior cerebral arteries. It is unusual for occipital infarction in the posterior cerebral artery (PCA) territory to be caused by internal carotid artery disease. The following describes a patient with this unusual presentation and highlights the mechanism of such a stroke and its implications.

A 22 year old male came to hospital complaining of headache, neck pain, and sudden onset left-sided hemianopia, upper limb paresis, and loss of sensation for a day. He had sustained blunt trauma to the head which involved neck hyper-extension, a week ago. He was discharged and was symptom free for approximately a week. Examination revealed a Glasgore Coma Score (GCS) of 15, left homonymous hemi-anopia, and left upper limb spastic mono-paresis with sensory loss. The rest of the examination was unremarkable.

**Figure 1:**
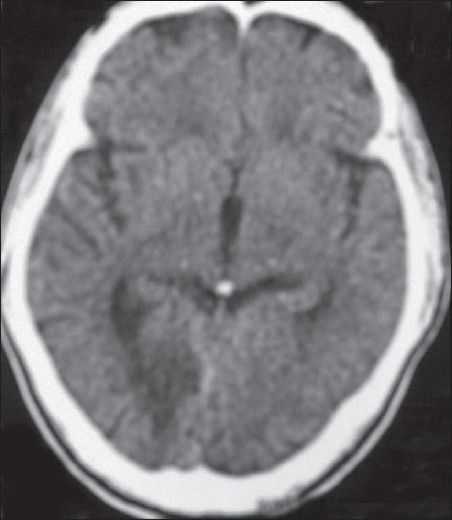
CT brain – right occipital infarct

**Figure 2:**
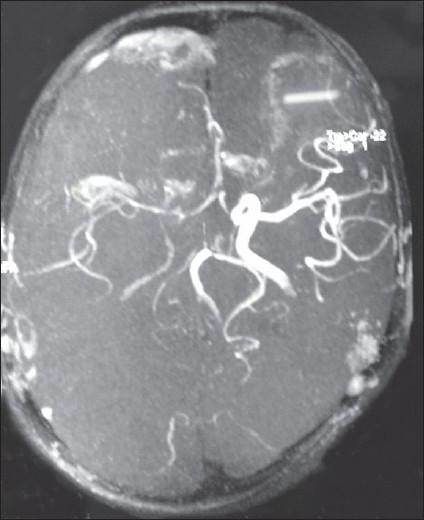
MR angiography–absent right PCA with adequate flow to the anterior and middle cerebral via ant. communicating artery

**Figure 3:**
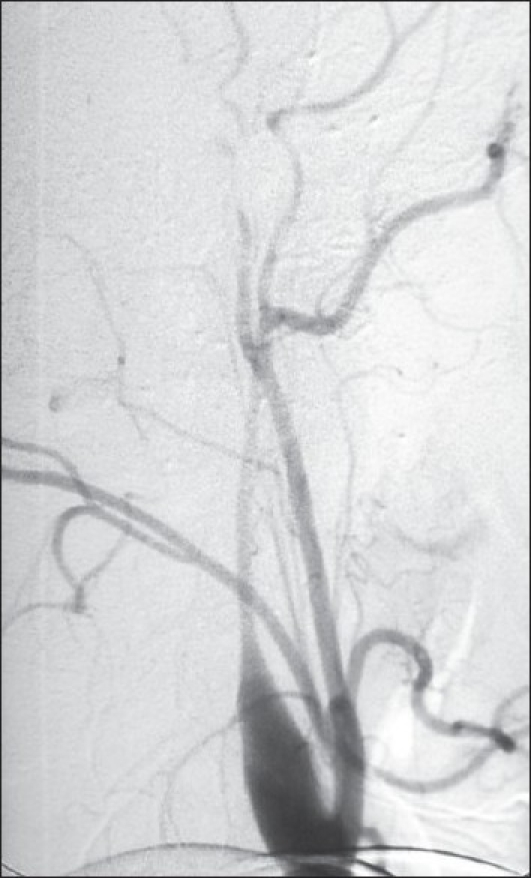
Carotid angiography–right internal carotid artery occlusion in the cervical region secondary to dissection

CT brain showed a right occipital infarct [Figure 1]. Magnetic resonance angiography (MRA) showed an absent right posterior cerebral artery (PCA) [Figure 2]. All other intracranial vessels were normal. Digital subtraction angiography demonstrated occlusion of the right cervical internal carotid artery due to dissection [Figure 3] and normal flow in all other extra cranial vessels. Cardiac source of embolism and thrombophilc states were excluded. The patient was anticoagulated.

Traumatic carotid artery dissection is a recognized complication following head injury and trauma to the neck. The mechanism of injury involves hyper-extension of the neck resulting in tethering of the internal carotid at the base of the skull or stretching over the lateral processes of the upper cervical vertebrae.[4] This results in the tearing of the vessel between adventia and intima or intima and media. Rupture through the intima causes the formation of thrombus within the lumen and may lead to a critical occlusion of the internal carotid artery or a thrombo-embolic episode. CAD on angiography shows irregular, and often tapered, stenosis with the characteristic "string sign," "flame-shaped" occlusion [Figure 3].

Symptoms in blunt carotid injury classically do not appear until 12 to 24 h after injury. Neurologic symptoms sometimes may be delayed for several weeks[5] The clinical picture of CAD is variable, with either pure local symptoms, such as Horner syndrome associated with headache or cervical pain, or cerebral ischemia in up to 80% of patients.[6]

Incidence studies suggest PCA territory infarcts to be more common among strokes in the young.[7] This patient presented with hemiplegia and hemsensory loss which are features of middle cerebral artery territory infarction, along with visual field defects. Previous studies have shown that 17% of patients with pure cortical PCA strokes have face-arm-leg motor deficits and 23% have sensory deficits in the same distribution.[8]

This patient's imaging demonstrates a right CAD absent right PCA [Figure 2] and an infarct in the right PCA territory [Figure 1]. Such an occurrence is possible when a persistent fetal PCA, which takes direct origin from the internal carotid artery, acts as a conduit for embolism from the anterior circulation. The adult configuration PCAs branch off the top of the basilar artery and supply parts of the midbrain, subthalamic and basal nuclei, thalamus, inferior temporal lobe, and occipitoparietal cortices. The PCA is divided in to two parts by the posterior communicating artery (PcomA), the proximal part is named as the pre-communicating part (P1) and the distal part as the post-communicating part (P2). In the fetal PCA, the diameter of the ipsilateral pre-communicating (P1) segment of PCA is less than PcomA, so that the blood supply to the occipital lobe is mainly via the internal carotid arteries. Fetal PCA arising predominantly from the ICA is seen in 4.4% of the Sri Lankan population, while in India the incidence remains as high as 25%.[9] This mechanism of stroke has been described.[10]

Traumatic CADs are often missed, as there is a delay in the onset of signs and symptoms and when these signs do develop, they are often attributed to an associated head injury rather than a vascular injury. Failure to make a diagnosis of CAD may result in long-term neurologic sequelae or death, although spontaneous resolution of the condition does sometimes occur.
